# Right Atrial Perforation With Pericardial Effusion and Visceral Pericardial Flap Observed During Pacemaker Implantation: An Echocardiographic Case Report

**DOI:** 10.7759/cureus.99361

**Published:** 2025-12-16

**Authors:** Frank Jorge Valdez Baez, Cemirame Payan Jimenez, Catherine Merejo Peña, Juanico Cedano Ramirez, Oscar Ortega, Donaldo Collado, Luisa Paulino Lopez, Warenny Montero Morillo, Evelina Severino Marte, Jonathan Rodriguez

**Affiliations:** 1 Electrophysiology, Asociación Instituto Dominicano de Cardiología, Santo Domingo, DOM; 2 Echocardiography, Asociación Instituto Dominicano de Cardiología, Santo Domingo, DOM; 3 Echocardiography, Cardio Imágenes Especializadas, Santo Domingo, DOM; 4 Imaging and Computed Tomography, Asociación Instituto Dominicano de Cardiología, Santo Domingo, DOM; 5 Anesthesia, Asociación Instituto Dominicano de Cardiología, Santo Domingo, DOM; 6 Cardiology Residency Program, Asociación Instituto Dominicano de Cardiología, Santo Domingo, DOM

**Keywords:** dual-chamber pacemaker, echocardiography, pacemaker implantation complications, pericardial effusion, right atrial perforation, subepicardial hematoma, visceral pericardial flap

## Abstract

Pacemaker implantation is a common procedure for the treatment of symptomatic bradyarrhythmias. Although generally safe, it can be associated with serious complications, such as cardiac perforation or pericardial effusion. However, right atrial wall perforation, accompanied by pericardial effusion and visualization of a visceral pericardial flap during the procedure, is an extremely rare and scarcely documented event.

We report the case of a 92-year-old woman with sinus node dysfunction who developed acute hypotension during dual-chamber pacemaker implantation. Echocardiography revealed findings consistent with right atrial perforation and pericardial effusion, which were confirmed by non-contrast computed tomography. The patient evolved favorably under conservative, non-surgical management. This case illustrates an uncommon complication of pacemaker implantation and underscores the importance of immediate hemodynamic and imaging assessment during acute intraoperative events.

## Introduction

The implantation of cardiac electronic devices, such as permanent pacemakers, represents a fundamental therapy in the management of cardiac rhythm disorders [1]. Although the procedure has a high safety profile, lead-induced cardiac perforation remains an uncommon but potentially serious complication [2]. The reported incidence of cardiac perforation associated with pacemaker implantation varies widely in the literature, ranging from 0.1% to 7%, depending on the diagnostic criteria and imaging modalities used [3,4]. This complication may present acutely, subacutely, or in a delayed fashion [5], and its severity depends on the extent of the injury, the location of the perforation, and the patient’s hemodynamic stability.

The most clinically relevant consequences include loss of capture, pericardial effusion, and cardiac tamponade, the latter being a life-threatening condition that often requires pericardiocentesis [1]. Management depends on the severity and hemodynamic impact of the event, ranging from conservative observation in stable patients [5] to lead extraction, repositioning, or open surgical repair in cases of frank perforation with circulatory compromise [6]. Other relevant mechanical complications include pneumothorax, which is particularly associated with subclavian access.

Reports of right atrial perforation with direct visualization of a visceral pericardial flap are extremely scarce, and diagnostic criteria for this finding remain poorly defined. Recognizing this imaging pattern is crucial for determining whether urgent surgical intervention is required or whether conservative management is appropriate.

## Case presentation

A 92-year-old woman with a history of arterial hypertension, type 2 diabetes mellitus, cerebrovascular accident, and chronic kidney disease, not requiring dialysis for the past three years, presented to the Emergency Department with recurrent syncopal episodes associated with convulsive movements, lasting approximately 10 days. She provided a 24-hour Holter recording for evaluation. Her medications included losartan 50 mg, amlodipine 10 mg, hydrochlorothiazide 25 mg, clopidogrel 75 mg, and carbamazepine.

**Figure 1:**
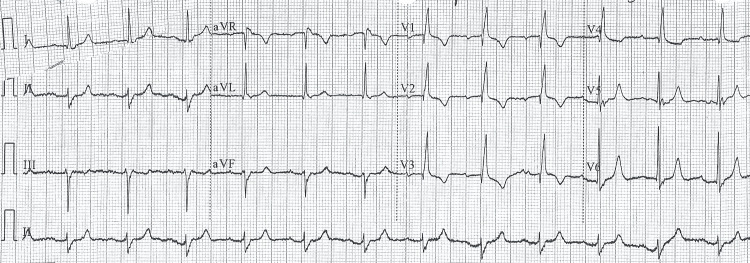
Baseline Electrocardiogram A 12-lead electrocardiogram showing sinus rhythm with a normal PR interval and a widened QRS complex. A complete right bundle branch block and left-axis deviation are present, findings consistent with left anterior fascicular block. No evidence of acute ischemia.

The baseline electrocardiogram (ECG) showed sinus rhythm, with a normal PR interval and a widened QRS complex. There was a complete right bundle branch block and left axis deviation, findings consistent with left anterior fascicular block. No signs of acute ischemia were observed (Figure 1).

**Figure 2:**
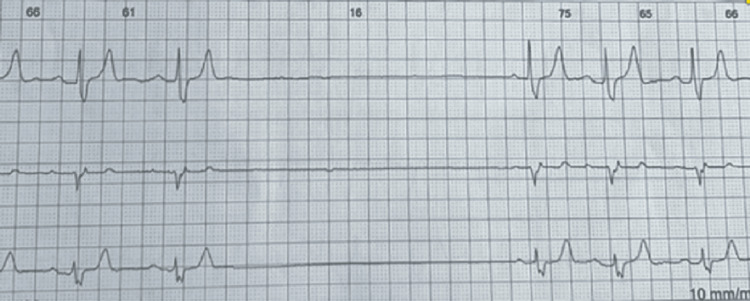
Twenty-Four-Hour Holter Recording Simultaneous three-lead tracing (leads II, V1, and V5) showing baseline sinus rhythm interrupted by prolonged sinus arrest episodes, with pauses exceeding 3.5 seconds and no escape activity - findings consistent with severe sinus node dysfunction. The QRS complexes display morphology consistent with complete right bundle branch block.

The 24-hour Holter recording revealed a baseline sinus rhythm, with prolonged sinus arrest episodes, including pauses longer than four seconds without escape activity, consistent with severe sinus node dysfunction. The QRS complexes demonstrated a morphology suggestive of complete right bundle branch block (Figure 2).

Procedure description

Temporary transvenous pacing was initiated, followed by the planned implantation of a permanent dual-chamber pacemaker. After standard sterile preparation of the operative field and administration of local anesthesia, an incision was made in the left subclavian region. Layer-by-layer dissection was performed to expose the muscular fascia, and a subcutaneous pocket was created to accommodate the pulse generator. Venous access was obtained via the subclavian vein using the Seldinger technique, after which introducers were advanced, and the pacing leads were positioned within the cardiac chambers. The ventricular and atrial leads were placed in the right ventricle and right atrium, respectively, with appropriate positioning confirmed fluoroscopically, and electrical parameters recorded. No technical difficulties were encountered during the procedure.

The ventricular lead demonstrated an R-wave amplitude of 7.4 millivolts (mV), impedance of 653 ohms, and a pacing threshold of 0.4 volts (V), whereas the atrial lead showed a P-wave amplitude of 3.1 mV, impedance of 482 ohms, and a threshold of 0.8 V. The implanted generator was manufactured by Shree Pacetronix, Pithampur, India, model Charak 747 Pro MR, a dual-chamber, magnetic resonance imaging (MRI)-compatible device. Both the atrial and ventricular leads were active-fixation type from the same manufacturer (model 3744VB), measuring 53 cm and 58 cm in length, respectively.

At the end of the procedure, the patient developed severe arterial hypotension (74/20 mmHg), which required fluid resuscitation and vasopressor support. A fluoroscopic review ruled out pneumothorax, and an immediate echocardiographic examination was performed to evaluate potential mechanical complications.

An immediate transthoracic echocardiogram (TTE) was performed in the electrophysiology laboratory using a portable Terason t3200 system (Teratech Corporation, Burlington, MA, USA). Images were acquired primarily in the subcostal four-chamber view and demonstrated the following findings (Video 1). A mild pericardial effusion was observed, represented by a thin anechoic layer partially surrounding the cardiac chambers - more evident posteriorly - without evidence of right-sided chamber collapse.

**Video 1:** Transthoracic Echocardiogram (Subcostal Four-Chamber View) Obtained During Pacemaker Implantation Transthoracic echocardiogram (subcostal four-chamber view) obtained during pacemaker implantation, demonstrating a mild pericardial effusion without right-sided chamber collapse, and a mobile echogenic flap adjacent to the right atrial wall, consistent with displacement of the visceral pericardium by a contained subepicardial hematoma. Left-sided chambers show preserved global function, with no evidence of cardiac tamponade.

In addition, a mobile echogenic lamina was identified within the pericardial space, corresponding to a slender and flexible linear structure running parallel to the right atrial and ventricular walls, exhibiting excursion synchronous with the cardiac cycle. Its morphology was consistent with the visceral pericardium displaced by an underlying collection or contained hematoma, rather than a fibrin strand or mural pseudoaneurysm. The spatial relationship to the atrial lead suggested a mural origin, adjacent to the lead anchoring site.

The right atrium maintained its normal anatomic configuration, without significant compression or collapse. The left ventricle demonstrated preserved global contractility, mild eccentric hypertrophy, and no significant dilation. No signs of cardiac tamponade or diastolic chamber collapse were observed, and valvular motion appeared normal in all acquired views.

The conclusion is: findings were consistent with displacement of the visceral pericardium by a contained subepicardial hematoma or collection, without evidence of right atrial dissection. After hemodynamic stabilization, the patient was transferred to the postoperative intensive care unit for observation and monitoring.

In-hospital course

During hospitalization, the patient remained hemodynamically stable and asymptomatic, without chest pain or dyspnea. A progressive decline in hemoglobin was observed, decreasing from 11.2 g/dL at admission to 9.3 g/dL, accompanied by a parallel reduction in hematocrit from 32.7% to 27.4%. This prompted a blood transfusion, after which hematologic parameters stabilized. The platelet count gradually decreased from 284 × 10³/µL to 172 × 10³/µL, without clinical evidence of active bleeding, while mild leukocytosis (10.68 × 10³/µL) was interpreted as a postoperative inflammatory response. Serum creatinine remained stable (1.62-1.87 mg/dL), and potassium levels, initially elevated at 6.12 mEq/L, normalized following medical management. Sodium levels remained within the physiological range (Table 1).

**Table 1:** Key Laboratory and Hemodynamic Parameters During Hospital Course Summary of the patient’s main laboratory and hemodynamic parameters from admission through the postoperative course, highlighting the transient anemia and hyperkalemia that stabilized with medical management.

Parameter	Admission	Post-implant	Post-implant (24 h)	Pre-discharge
Hemoglobin (g/dL)	11.2	-	9.3	9.3 (after transfusion)
Hematocrit (%)	32.7	-	27.4	27.4
Platelets (×10³/µL)	284	-	172	172
Creatinine (mg/dL)	1.62	-	1.87	1.70
Potassium (mEq/L)	6.12	5.2	4.3	4.4
Blood Pressure (mmHg)	118/70	74/20 (hypotension)	110/68	120/70

**Figure 3:**
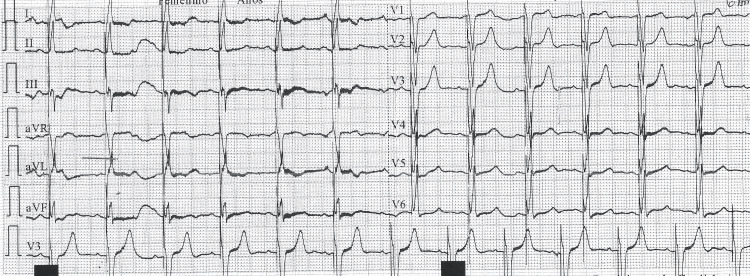
Post-implantation Electrocardiogram Regular rhythm at 82 bpm with sinus P waves preceding each QRS complex, indicating effective atrial sensing. Pacemaker spikes precede each QRS, consistent with dual-chamber pacing in DDD mode. The QRS complex is widened with morphology of an artificial left bundle branch block and an electrical axis of approximately +30°, suggestive of septal right ventricular pacing.

The post-implant ECG (Figure 3) showed a regular rhythm at 82 bpm, with sinus P waves preceding each QRS complex, indicating effective atrial sensing. Pacemaker spikes preceded every QRS complex, with no visible atrial spikes - findings consistent with a dual-chamber (DDD) pacing mode featuring atrial sensing and ventricular pacing. The QRS complexes were widened, displaying a morphology compatible with an artificial left bundle branch block pattern. The frontal QRS axis was approximately +30°, suggesting right ventricular septal stimulation. No acute ischemic changes were observed, and the electrical function of the device was appropriate.

**Figure 4:**
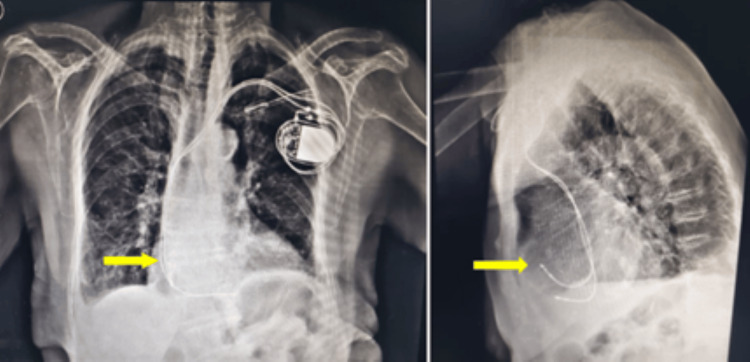
Posteroanterior and Lateral Chest Radiographs Chest radiographs (posteroanterior and lateral views) showing the pacemaker generator in the left subclavicular position with leads correctly positioned in the right heart chambers - the atrial lead anchored to the anterolateral wall of the right atrium and the ventricular lead directed toward the septal or apical region of the right ventricle. The cardiac silhouette appears mildly enlarged, with elongation and calcification of the aortic arch, increased bronchovascular markings, and a subtle basal interstitial pattern. No evidence of pneumothorax or generator displacement (arrows indicate the anchoring site of the right atrial lead).

Chest radiographs in posteroanterior and lateral projections (Figure 4) show the pacemaker generator in the left subclavicular region, with leads appropriately positioned within the right-sided cardiac chambers: the atrial lead anchored to the anterolateral wall of the right atrium, and the ventricular lead directed toward the septal or apical region of the right ventricle. The cardiac silhouette is mildly enlarged, with a cardiothoracic ratio at the upper limit of normal. The aortic arch appears elongated and sclerotic, with parietal calcifications consistent with chronic aortic atheromatosis. The pulmonary parenchyma demonstrates diffuse prominence of the bronchovascular markings and a subtle basal reticular pattern, more pronounced in the right hemithorax, suggestive of mild interstitial lung disease and a small right-sided pleural effusion. No pulmonary infiltrates, masses, or pneumothorax are evident. The bony structures reveal diffuse osteopenia, thoracic spondylosis with increased kyphosis, and bilateral acromioclavicular degenerative changes, with no signs of fracture or acute osseous injury.

The TTE performed 18 hours after implantation showed eccentric left ventricular hypertrophy, with septal and posterior wall thickness of 0.89 cm, and preserved ejection fraction (left ventricular ejection fraction (LVEF) 54%, Simpson’s method). The left atrium was severely dilated (volume ≈ 65 mL). A double mitral lesion was observed, consisting of mild stenosis and moderate regurgitation, along with mild aortic and tricuspid regurgitation. The tricuspid annular plane systolic excursion (TAPSE) measured 23 mm, indicating preserved right ventricular systolic function. A small posterior pericardial effusion was present, without right-sided chamber collapse or signs of cardiac tamponade. Throughout this period, the patient remained asymptomatic and clinically stable.

**Figure 5:**
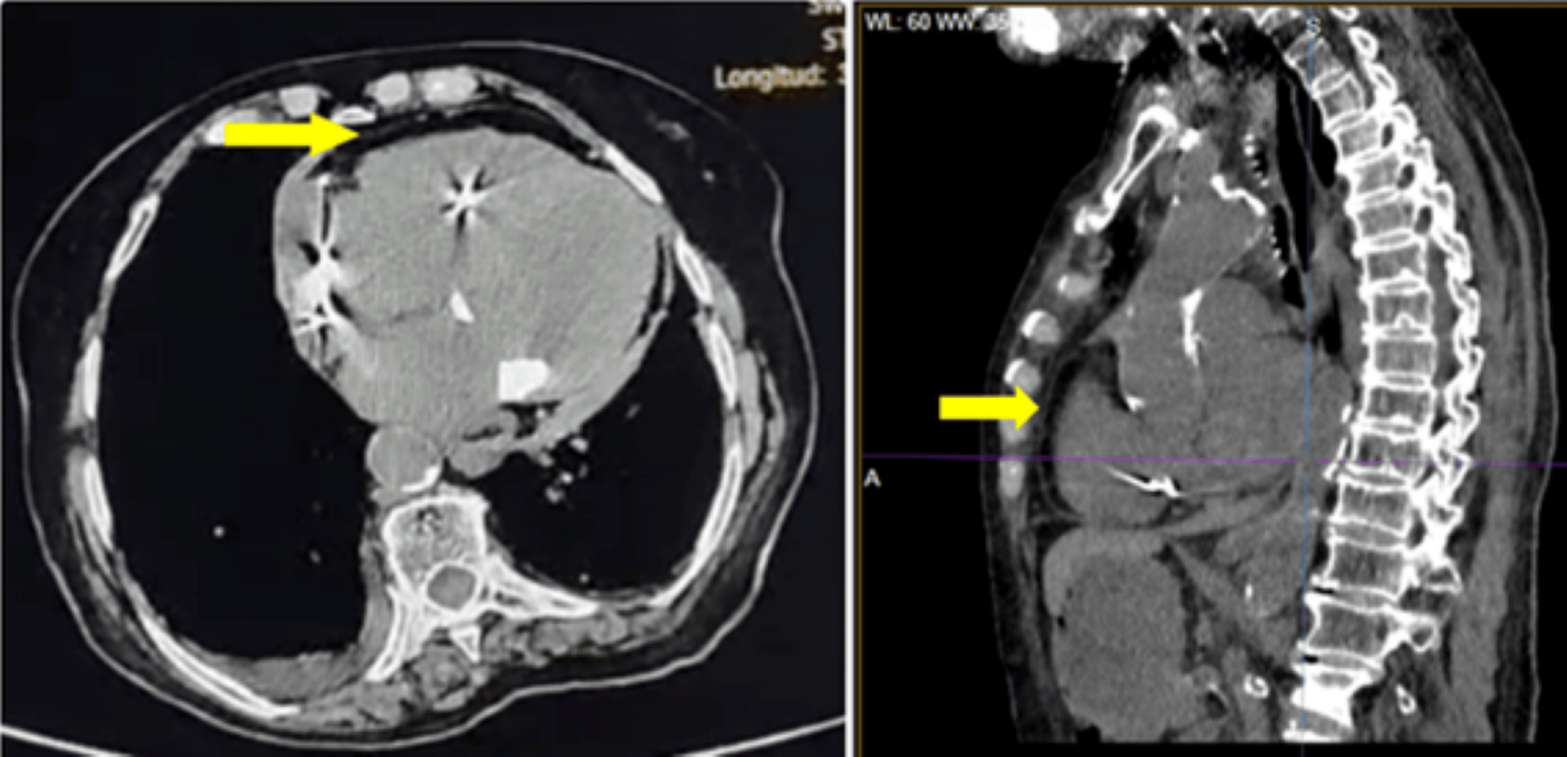
Non-contrast Chest Computed Tomography Axial (left) and sagittal (right) computed tomography images demonstrate a high-attenuation pericardial effusion consistent with mild hemopericardium (arrow) and focal mural heterogeneity along the anterior right atrial wall (arrow), suggestive of a contained microhematoma at the lead fixation site. Additional findings include a small-to-moderate right pleural effusion, diffuse coronary and aortic calcifications, and subtle basal interstitial thickening consistent with mild interstitial lung disease.

The non-contrast chest computed tomography (CT) performed 24 hours after implantation (Figure 5) revealed an area of mural heterogeneity in the anterior wall of the right atrium, partially obscured by metallic artifacts from the lead, consistent with a contained microhematoma at the atrial fixation site. A small pericardial effusion with hematic density (31 Hounsfield units) was noted, without evidence of active extravasation or cardiac tamponade. Additional findings included dilation of the main pulmonary artery, mitral annular calcification, and diffuse atherosclerotic changes involving the coronary arteries and aorta. Mild subpleural bronchiectasis and interstitial thickening were present, without consolidations or masses. A small-to-moderate right-sided pleural effusion was also identified. Overall, the findings were consistent with a mild hemopericardium, secondary to a contained microlesion of the right atrial wall, without evidence of free perforation or hemodynamic compromise.

A two-dimensional echocardiogram performed eight days after the event confirmed only a small pericardial effusion and an LVEF of 65%, which allowed hospital discharge in an asymptomatic and hemodynamically stable condition.

**Figure 6:**
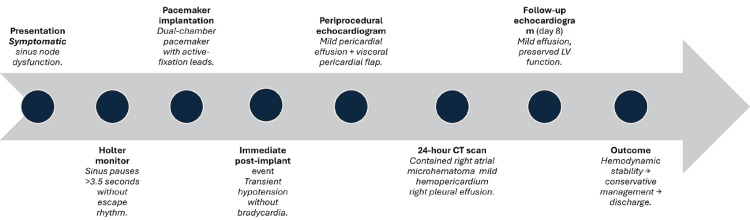
Clinical Timeline of Event Chronological sequence summarizing the patient’s clinical course, beginning with symptomatic sinus node dysfunction and Holter documentation of sinus pauses, followed by dual-chamber pacemaker implantation and a transient post-procedural hypotensive episode. Periprocedural echocardiography revealed a mild pericardial effusion with a mobile visceral pericardial flap, and a 24-hour CT scan confirmed a contained right atrial microhematoma with mild hemopericardium and a small right pleural effusion. A follow-up echocardiogram on day 8 demonstrated a stable mild effusion and preserved ventricular function, supporting successful conservative management and discharge.

In summary, the patient presented with a contained perforation of the right atrium, associated with a mild pericardial effusion, without evidence of cardiac tamponade or pacemaker capture loss. Clinical stability, normal device function, and the absence of effusion progression confirmed the limited nature of the event and justified a conservative management approach. The integration of echocardiographic and tomographic imaging was essential for characterizing the lesion and guiding therapeutic decisions, ensuring a favorable outcome without complications. A detailed chronological sequence of events is illustrated in the clinical timeline (Figure 6).

## Discussion

Rarity and localization of the right atrial lesion

Right atrial perforation during pacemaker implantation represents an exceptional complication within the spectrum of mechanical events associated with this procedure. In contemporary series, most perforations involve the right ventricle, with reported incidences ranging between 0.1% and 0.5% [1]. Atrial involvement is even rarer and, when it occurs, is typically associated with active-fixation leads positioned in the right atrial appendage or interatrial septum [7,8].

The echocardiographic finding of a mobile flap adjacent to the right atrial wall in our patient most likely corresponds to displacement of the visceral pericardium by a localized subepicardial hematoma, secondary to focal microperforation during active lead fixation. This interpretation is supported by the synchronous motion of the flap with the cardiac cycle and the absence of Doppler flow across the atrial wall - features that exclude free perforation or mural dissection.

Anatomically, the right atrium lies in close relation to the atrioventricular groove and the epicardial course of the right coronary artery. This proximity explains how active-fixation leads, placed along the anterolateral or inferior atrial wall, may cause microinjury to small epicardial branches or superficial coronary veins. In elderly patients, myocardial atrophy and interstitial fibrosis reduce tissue resistance, predisposing to limited subepicardial bleeding that may separate the visceral pericardium and generate a mobile echogenic flap.

In this context, Nakajima et al. [8] reported a case in which an active-fixation atrial lead perforated the right atrial wall and reached the ascending aorta, resulting in delayed cardiac tamponade. Although the clinical outcome was more severe, both cases emphasize the anatomical vulnerability of the right atrial wall and its proximity to the right coronary artery.

In our patient, preservation of atrial capture and hemodynamic stability suggests that the injury remained confined, without significant transmural involvement. Altogether, age-related tissue fragility, focal lead trauma, and possible epicardial microvascular injury constitute a plausible pathophysiological mechanism for the development of the subepicardial hematoma and visceral pericardial displacement observed.

Hemodynamic instability and conservative management

From a clinical standpoint, the hemodynamic impact of contained atrial lesions can vary widely. The transient hypotension observed in the absence of cardiac tamponade may be explained by a combination of reflex and hemodynamic mechanisms. A vagal reflex, triggered by mechanical stimulation of the atrial wall or visceral pericardium by the lead, may induce transient bradycardia and hypotension - a phenomenon previously described during intracardiac procedures and attributed to activation of pericardial mechanoreceptors, which typically resolves once the stimulus ceases. Additionally, formation of a contained subepicardial microhematoma may have produced localized compression of the right atrium or venous return, transiently reducing preload. The proximity of the right coronary artery also raises the possibility of minor epicardial microvascular compromise or limited extravasation, sufficient to explain hypotension without progression to global tamponade. Collectively, these reflex-hemodynamic mechanisms provide a coherent explanation for the brief hypotensive episode and the patient’s spontaneous recovery.

Preservation of atrial sensing and pacing, despite perforation, indicates that the active-fixation lead maintained effective contact with viable myocardium, allowing normal electrical conduction. In contained microperforations, the helix may partially traverse the atrial wall without losing continuity with excitable fibers, thereby maintaining stable thresholds and sensing signals. Moreover, the adjacent subepicardial hematoma or localized collection may act as a partial electrical insulator, without significantly altering impedance, which explains the stable pacemaker function and absence of capture loss despite localized structural compromise.

In previous reports, conservative management has proven effective in hemodynamically stable patients with limited perforations and preserved capture [1,5]. Partial perforations, with minimal extravasation - whether atrial or ventricular - have demonstrated spontaneous resolution under close observation and temporary suspension of anticoagulation therapy. Conversely, hemodynamic instability with cardiac tamponade necessitates urgent drainage or surgical repair, as described in cases of ventricular perforation complicated by hemothorax or intercostal artery injury [9,10]. The favorable outcome in our patient, without the need for lead extraction or surgery, reinforces the principle that the extent of bleeding and clinical stability should guide management decisions, avoiding unnecessary invasive procedures in contained lesions.

Diagnostic value of echocardiography and CT

The timely diagnosis of mechanical complications related to pacemaker implantation depends on the integration of multiple imaging modalities. In this case, transthoracic echocardiography identified a mobile echogenic flap adjacent to the right atrial wall, corresponding to the visceral pericardium displaced by a contained subepicardial hematoma, along with a mild pericardial effusion. Non-contrast chest CT further demonstrated mural heterogeneity and confirmed the hemorrhagic density of the pericardial fluid, findings consistent with a contained lesion.

Recent series emphasize that the combined use of echocardiography and CT is essential for differentiating between free, intramural, or lead-displacement perforations [11,12]. Point-of-care ultrasound (POCUS) has emerged as a valuable early diagnostic tool, allowing bedside recognition of characteristic echocardiographic findings, such as the “bow-and-arrow sign,” which aids in identifying atrial or apical perforations [11]. Likewise, cardiac CT enables confirmation of more complex cases, including lead migration from the right ventricle into left-sided chambers or great vessels - events reported even as causes of delayed cardiac tamponade [8,12].

These observations support the multimodal imaging strategy adopted in our patient, where the integration of echocardiography and CT enabled precise characterization of a contained event, thereby avoiding diagnostic misinterpretation and unnecessary invasive interventions.

Therapeutic approach and individualized decision-making

Accurate diagnosis directly determines the therapeutic strategy. The management of lead-related perforations varies according to their anatomical location and clinical impact. In hemodynamically stable patients with contained lesions, close observation and temporary discontinuation of anticoagulation are considered safe strategies [13]. In contrast, free perforations involving the ventricular wall or great vessels require immediate lead extraction or urgent surgical intervention [14].

The present case belongs to a minority group of patients successfully managed conservatively, in whom the lesion did not compromise electrical function or the structural integrity of the pacing system. This approach contrasts with reports of septal or ventricular perforations treated surgically through direct suture and lead repositioning [13,14].

The favorable outcome, without invasive intervention, reinforces the principle that therapy should be individualized, guided by clinical stability, effusion size, and multimodal imaging findings. Confirmation of a contained lesion, without signs of tamponade, allows the clinician to avoid premature lead extraction, which may otherwise exacerbate structural injury and increase the risk of bleeding or hemodynamic instability.

A direct comparison between conservative and surgical approaches highlights the factors that favored non-operative management in this case. Surgical extraction or repair is generally indicated in patients with hemodynamic instability, large or expanding pericardial effusions, loss of capture, or evidence of free perforation. In contrast, our patient exhibited none of these high-risk features. The combination of hemodynamic stability, a small, non-progressive pericardial effusion, and preserved atrial sensing and capture supported a conservative approach with close monitoring. These simple criteria are consistent with previously reported favorable outcomes in contained atrial or ventricular microperforations, in which conservative management has proven safe and effective.

Limitations of the case

This case report presents several limitations inherent to the clinical context. The initial echocardiogram was performed under suboptimal technical conditions, which may have limited structural definition. Although color Doppler assessment was conducted to rule out flow across the atrial wall, it was not captured on video; however, direct observation revealed no flow through the lesion. Both the chest CT and the formal TTE were obtained 18-24 hours after the initial event, and may therefore not fully represent the immediate findings. In addition, the CT study was performed without contrast due to the patient’s impaired renal function, limiting vascular assessment and the detailed characterization of the subepicardial hematoma.

An additional limitation is that several echocardiographic descriptors - such as the degree of pericardial effusion and the severity of valvular regurgitation - were reported qualitatively rather than quantitatively in the formal echocardiographic study. Because specific numerical measurements (e.g., effusion thickness in millimeters, vena contracta width, or valve gradients) were not included in the final report, certain findings could not be converted into objective values and had to be presented using standard qualitative terminology. This subjective nature of the source report may limit precision in the characterization of some structural abnormalities.

Nevertheless, these limitations did not preclude the diagnosis of a contained right atrial perforation or affect the overall clinical and imaging interpretation of the case.

## Conclusions

This case expands the clinical spectrum of complications associated with pacemaker implantation by incorporating a contained right atrial perforation as an exceptionally rare manifestation. It also highlights the importance of immediate echocardiographic evaluation following post-implant hypotensive episodes, even in the absence of tamponade, and demonstrates that multimodal imaging integration is essential for distinguishing contained lesions, amenable to conservative management. Comparison with previously reported cases confirms that right atrial involvement is extraordinary, and that a non-surgical approach can be both safe and effective when the diagnosis is established early through complementary imaging techniques.
